# Rat Strain Differences in Susceptibility to Alcohol-Induced Chronic Liver Injury and Hepatic Insulin Resistance

**DOI:** 10.1155/2010/312790

**Published:** 2010-08-16

**Authors:** Sarah M. DeNucci, Ming Tong, Lisa Longato, Margot Lawton, Mashiko Setshedi, Rolf I. Carlson, Jack R. Wands, Suzanne M. de la Monte

**Affiliations:** Departments of Medicine and Pathology, Liver Research Center, Rhode Island Hospital and the Warren Alpert Medical School of Brown University, Pierre Galletti Research Building, 55 Claverick Street, Room 421, Providence, RI 02903, USA

## Abstract

The finding of more severe steatohepatitis in alcohol fed Long Evans (LE) compared with Sprague Dawley (SD) and Fisher 344 (FS) rats prompted us to determine whether host factors related to alcohol metabolism, inflammation, and insulin/IGF signaling predict proneness to alcohol-mediated liver injury. Adult FS, SD, and LE rats were fed liquid diets containing 0% or 37% (calories) ethanol for 8 weeks. Among controls, LE rats had significantly higher ALT and reduced GAPDH relative to SD and FS rats. Among ethanol-fed rats, despite similar blood alcohol levels, LE rats had more pronounced steatohepatitis and fibrosis, higher levels of ALT, DNA damage, pro-inflammatory cytokines, ADH, ALDH, catalase, GFAP, desmin, and collagen expression, and reduced insulin receptor binding relative to FS rats. Ethanol-exposed SD rats had intermediate degrees of steatohepatitis, increased ALT, ADH and profibrogenesis gene expression, and suppressed insulin receptor binding and GAPDH expression, while pro-inflammatory cytokines were similarly increased as in LE rats. Ethanol feeding in FS rats only reduced IL-6, ALDH1–3, CYP2E1, and GAPDH expression in liver. In conclusion, susceptibility to chronic steatohepatitis may be driven by factors related to efficiency of ethanol metabolism and degree to which ethanol exposure causes hepatic insulin resistance and cytokine activation.

## 1. Introduction

Chronic alcohol abuse causes liver injury that can progress through stages of simple steatosis to steatohepatitis, fibrosis, cirrhosis, and finally end-stage liver disease, and it also impairs the regenerative capacity of the liver [1–4]. Ethanol mediates these effects by compromising insulin signaling, which is needed for DNA synthesis, cell survival, gene expression, cell motility, and energy metabolism [5–10]. Insulin functions by activating its own receptor tyrosine kinase, which transmits signals downstream through insulin receptor substrate proteins and a complex sequence of adaptor molecules [11]. Ethanol inhibits at all levels of the insulin signaling cascade, but importantly, it impairs insulin receptor binding [12, 13] and activation of insulin receptor tyrosine kinase [6–9, 14]. Consequently, the toxic/metabolic effects of ethanol are mediated at proximal points within the insulin signaling cascade [14, 15], thereby accounting for the broad range of cellular functions impaired by ethanol [11], and the varied mechanisms of liver injury including increased DNA damage, activation of proapoptosis pathways, and mitochondrial dysfunction [14, 16, 17].

Previous studies showed that Long Evans (LE) rats were highly susceptible to the effects of chronic alcohol exposure, both in terms of liver [14] and brain [18–21] injury. In contrast to other rodent models in which chronic ethanol feeding for as long as 30 or 40 weeks results in mild microsteatosis with minimal inflammation, LE rats develop prominent steatohepatitis with increased DNA damage and evidence of apoptosis and necrosis within 5 or 6 weeks of ethanol feeding [12, 14, 15, 22]. Moreover, chronic ethanol feeding in LE rats causes hepatic insulin resistance with inhibition of survival signaling [12]. Altogether, the abnormalities produced by chronic ethanol exposure in LE rats resemble human chronic alcoholic steatohepatitis, suggesting that this model is an excellent tool for exploring means of preventing or minimizing the long-term consequences of alcohol-induced liver injury, including deficits in the liver's capacity to regenerate. 

 Empirical observations in our laboratory revealed that chronic ethanol exposure in other strains, including Sprague Dawley (SD) and Fisher 344 (FS) rats, did not produce as robust a model of steatohepatitis compared with LE rats, raising a fundamental question about the role of genetic or strain background in relation to host susceptibility to alcohol-mediated liver injury. We hypothesized that premorbid and/or postethanol exposure differences in hepatic insulin responsiveness, alcohol metabolizing enzyme gene expression, and proinflammatory status in liver would correlate with severity of alcoholic steatohepatitis. To address this question, we compared liver histology and several biochemical and molecular parameters of hepatic dysfunction, including steatosis, insulin and insulin-like growth factor (IGF) receptor binding, and mRNA levels of alcohol metabolizing enzymes, proinflammatory cytokine, and hepatic cellular function genes among LE, SD, and FS rats which were empirically found to differ with respect to severity of chronic ethanol-induced liver injury. The primary objective of this study was to determine if the strains differed significantly with regard to these parameters, either before or after chronic ethanol exposure. The expectation was that if pre- or postethanol exposure differences exist and correlate with injury and functional impairments in liver, future efforts could be productively directed to identify relevant biomarkers and design therapies that target fundamental abnormalities.

## 2. Methods

### 2.1. Chronic Ethanol Exposure Model

Adult male (~200–250 g) FS, LE, and SD rats (Harlan Sprague Dawley, Inc., Indianapolis, Indiana) were pair-fed with isocaloric liquid diets (BioServ, Frenchtown, NJ) for 8 weeks [14]. Two weeks prior to the experiment, rats were adapted to the liquid diets by incrementing the ethanol content from 0% to 11.8%, 23.6%, and then 37% of the caloric content. Controls were adapted to ethanol-free liquid diets over the same period. Thereafter, rats were maintained on 0% or 37% ethanol-containing diets throughout the study [14]. Rats were monitored daily to ensure adequate nutritional intake and maintenance of body weight. Blood alcohol levels were measured at 8 AM using the Analox GM7 apparatus (Analox Instruments USA, Lunenburg, MA) according to the manufacturer's protocol. At the end of the feeding period, the rats were sacrificed by i.p. injection of 120 mg/kg sodium pentobarbital, and liver tissues were snap-frozen in a dry ice/methanol bath for later protein and RNA studies, or immersion fixed in Histochoice (Amresco, Solon, OH) for histological studies. Throughout the experiment, rats were housed under humane conditions and kept on a 12-hour light/dark cycle with free access to food. All experiments were performed in accordance with protocols approved by Institutional Animal Care and Use Committee at the Lifespan-Rhode Island Hospital, and conform to guidelines established by the National Institutes of Health. 

### 2.2. Histopathologic Studies

Paraffin-embedded histological sections were stained with Hematoxylin and Eosin (H&E), and adjacent sections were stained with Sirius Red F3B to detect fibrosis, or immunostained to detect single-stranded DNA as an index of DNA damage [23]. Prior to immunostaining, the tissue sections were pretreated with proteinase K and blocked to reduce nonspecific binding according to the manufacturer's protocol. The sections were immunostained with 1 *μ*g/mL of mouse monoclonal anti-single-stranded DNA antibody (Chemicon, Temecula, CA), and immunoreactivity was detected with horseradish peroxidase (HRP)-conjugated polymer-tagged secondary antibodies (Abcam, Cambridge, MA, USA) and diaminobenzidine as the chromogen. The sections were lightly counterstained with Hematoxylin. Percentages of labeled cells per 100x magnification field were determined using Image Pro-3 Plus software (Media Cybernetics Inc., Bethesda, MD).

### 2.3. Lipid Assays

 Lipid analyses were performed on chloroform-methanol (2 : 1) extracted fresh frozen liver tissue homogenates [24]. Total lipid content was measured using a Nile Red fluorescence-based assay (Molecular Probes, Eugene, OR) [25–27], and fluorescence intensity (Ex 485/Em 572) was measured in a SpectraMax M5 microplate reader (Molecular Devices Corp., Sunnyvale, CA). Triglycerides and cholesterol were measured with commercially available assay kits (Serum Triglyceride Determination kit, Sigma-Aldrich Co., St. Louis, MO; Amplex Red Cholesterol Assay Kit, Molecular Probes, Eugene, OR). Results were normalized to liver sample weight.

### 2.4. Quantitative Reverse Transcriptase Polymerase Chain Reaction (qRT-PCR) Assays of Gene Expression

Total RNA was reverse transcribed with random oligodeoxynucleotide primers, and the resulting cDNA templates were PCR amplified with gene specific primers [28] (Table 1). Amplified products were detected and analyzed in triplicate using the Mastercycler ep realplex instrument and software (Eppendorf AG, Hamburg, Germany) [24]. Relative mRNA abundance was calculated from the ng ratios of mRNA to 18S rRNA measured in the same samples, and those data were used for intergroup comparisons. Control studies included analysis of: (1) template-free reactions, (2) RNA that had not been reverse transcribed, (3) RNA samples pretreated with DNAse I, (4) samples treated with RNAse A prior to the reverse transcriptase reaction, and (5) genomic DNA. Although mRNA levels measured by qRT-PCR can be compared using various approaches, including the 2^−ΔΔCT^ method [29], we elected to calculate relative transcript abundance with 18S rRNA as the denominator because this approach allows one to more effectively correct for differences in template input and differences in amplification efficiency pertaining to rat strain and ethanol exposure [30].

### 2.5. Enzyme-Linked Immunosorbant Assay (ELISA)

Tissues were homogenized in radioimmunoprecipitation assay buffer containing protease and phosphatase inhibitors [31]. Direct binding ELISAs were performed in 96-well plates (Nunc, Rochester, NY) [18]. In brief, proteins (40 ng/100 *μ*L) adsorbed to well bottoms by overnight incubation at 4°C were blocked with 3% BSA in Tris buffered saline (TBS) and then incubated with primary antibody (0.2–1.0 *μ*g/mL) for 1 hour at room temperature. Immunoreactivity was detected with horseradish peroxidase- (HRP-) conjugated secondary antibody (1 : 10000; Pierce, Rockford, IL) and Amplex Red soluble fluorophore (Molecular Probes, Eugene, OR) [18]. Fluorescence was measured (Ex 530/Em 590) in a SpectraMax M5 microplate reader. Binding specificity was monitored in parallel control incubations that included nonrelevant antibodies, or omitted the primary or secondary antibody. Immunoreactivity was normalized to protein content in parallel wells as determined with the NanoOrange Protein Quantification Kit (Molecular Probes, Eugene, OR).

### 2.6. Receptor Binding Assays

Competitive equilibrium binding studies were used to measure effects of chronic ethanol feeding on insulin, IGF-1, and IGF-2 receptor binding [20, 31]. For total binding, NP-40 lysis buffer homogenates were incubated in 100 *μ*L reactions containing binding buffer (100 mM HEPES (4-(2-hydroxyethyl)-1-piperazine-ethanesulfonic acid), pH 8.0, 118 mM NaCl, 1.2 mM MgSO_4_, 8.8 mM dextrose, 5 mM KCl, 1% bovine serum albumin) and 0.0031 to 1 *μ*Ci/mL of [^125^I] (2000 Ci/mmol) 50 nCi/ml of [^125^I] (2000 Ci/mmol; 50 pM) insulin, IGF-1, or IGF-2. For nonspecific binding, identical reactions were prepared with the addition of 0.1 *μ*M unlabeled ligand. After 16-hours incubation at 4°C, reactions were harvested (Corning, Lowell, MA) onto 96-well GF/C filter plates that were presoaked in 0.33% polyethyleneimine. The filters were vacuum washed with 50 mM HEPES, pH 7.4, 500 mM NaCl, and 0.1% BSA. [^125^I]-bound insulin, IGF-1, or IGF-2 was measured in a TopCount (Perkin-Elmer, Boston, MA). Specific binding was calculated by subtracting fmol/mg of nonspecifically bound isotope from the total bound isotope. The results were plotted and analyzed using GraphPad Prism 5 software (San Diego, CA).

### 2.7. Sources of Reagents

Human recombinant [^125^I]-Insulin, IGF-1, and IGF-2 were purchased from Amersham Biosciences (Boston, MA). Unlabeled human insulin and recombinant IGF-1 and IGF-2 were obtained from Bachem (Torrance, CA). QuantiTect SYBR Green PCR Mix was obtained from Qiagen, Inc. (Valencia, CA). Unless otherwise indicated, all other fine chemicals were purchased from either CalBiochem (Carlsbad, CA) or Sigma-Aldrich (St. Louis, MO).

### 2.8. Statistical Analysis

Data depicted in the graphs and tables represent the means ± S.E.M.'s for 8–12 samples per group. Intergroup comparisons were made using two-way ANOVA and the Bonferroni post hoc multiple comparison test. Statistical analyses were performed using the GraphPad Prism 5 software (GraphPad Software, Inc., San Diego, CA). Significant *P*-values are shown in the graphs and tables. 

## 3. Results

### 3.1. Weight Gain Associated with Liquid Diet Feeding

**Figure 1:**
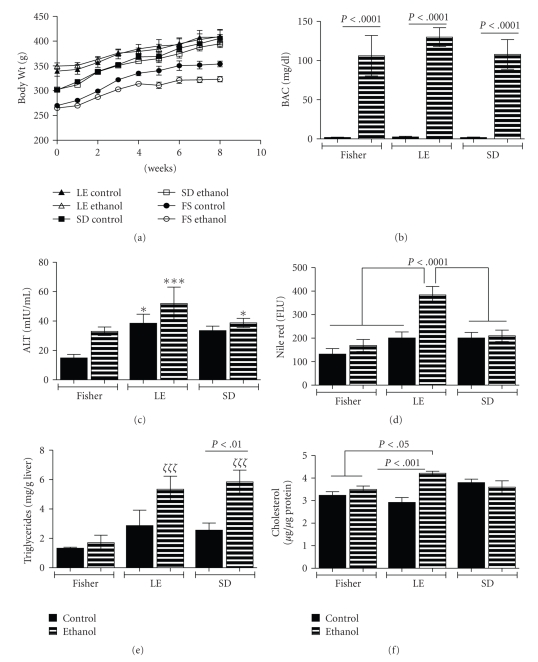
*Rat strain differences in effects of chronic alcohol feeding on weight gain, liver injury, and steatosis*: Fisher 344 (FS), Long Evans (LE), and Sprague Dawley (SD) rats were fed with isocaloric liquid diets containing 0% or 37% ethanol (caloric content) for 8 weeks. (a) Weekly changes in body weight are depicted graphically. (b) Blood alcohol concentrations and (c) serum ALT levels were measured at the time of sacrifice. (d) Nile Red fluorescence (neutral lipids), (e) triglycerides, and (f) cholesterol levels were measured in liver tissue, and results were normalized to sample weight or protein content. Graphs depict the mean ± S.E.M. Intergroup comparisons were made using ANOVA with the post hoc Bonferroni multiple comparisons test. Significant *P*-values are indicated within the panels. In addition, *P < .05 relative to FS control; ***P < .001 relative to FS control; *ζ* *ζ* *ζ* *P* < .001 relative to FS control and ethanol-fed rats.

 Rats in all groups consumed similar quantities of food, and correspondingly all gained weight over the course of the study. Despite identical birth weights and ages, FS rats had the lowest original and final body weights, SD rats were intermediate, and LE's were the largest. The curves corresponding to body weight over time were virtually identical for pair-fed LE rats and SD rats, whereas in FS rats, the trends were more tapered for ethanol-fed relative to controls after 3-4 weeks on the liquid diets (Figure 1(a)). Correspondingly, linear regression analysis of weight gain over time demonstrated slopes of 9.28 ± 1.49 and 7.77 ± 1.09 for control and ethanol-fed LE rats (*P* = .42), and 12.63 ± 0.80 and 11.39 ± 0.81 for control and ethanol-fed SD rats (*P* = .277), reflecting the similar within-group mean percentage increases in body weight (Table 2). In contrast, for the FS strain, the slopes reflecting weight gain over time were 11.18 ± 0.82 and 7.74 ± 0.68 for control and ethanol fed rats (*P* = .0018), correlating with the significant differences in their mean percentages of weight gained (Table 2). Among the 3 ethanol-fed groups, trends in weight gain were similar for the FS (Slope = 7.74 ± 0.67) and LE (Slope = 7.77 ± 1.09) strains, and both were significantly lower than that measured for the SD strain (11.39 ± 0.81) (*P* = .0037). Therefore, FS and LE rats exhibited similar percentage increases in body weight, but the ethanol-associated dampening of growth in FS rats reduced the net weight gained relative to the other strains. SD rats gained more weight and at faster rates than the other two groups. Despite these differences in weight gained over time, the mean blood alcohol levels were similar among the ethanol-fed groups (Figure 1(b)). Therefore, any distinctions found with respect to the effects of chronic ethanol exposure could not be attributed to differences in blood alcohol levels.

### 3.2. Effects of Ethanol on Biomarkers of Liver Injury and Steatosis

 Among controls, mean serum alanine transaminase (ALT) was significantly higher in LE compared with FS rats (Figure 1(c)), indicating higher baseline levels of hepatocellular injury in the LE strain. Among ethanol-fed rats, the LE strain also had the highest mean ALT. Although ethanol feeding increased ALT in all 3 strains relative to pair-fed controls, the within-group differences were not statistically significant due to variability in the magnitude of responses. However, mean ALT was significantly higher in ethanol-fed LE and SD rats than in FS controls. 

The Nile red assay was used to quantify hepatic neutral lipid content (fluorescent light units; FLU) with values normalized to sample weight (Figure 1(d)). Hepatic Nile red fluorescence was significantly increased by chronic ethanol exposure in the LE but not in the FS or SD strain. Moreover, Nile red fluorescence was significantly higher in livers of ethanol-fed LE rats compared to all other groups. 

Hepatic triglyceride content was similarly low among the 3 control groups, and the chronic ethanol-fed FS rats (Figure 1(e)). Chronic ethanol feeding significantly increased hepatic triglyceride content in LE and SD relative to control and ethanol-fed FS rats. In addition, hepatic triglyceride content was significantly higher in ethanol-fed relative to control SD rats. The mean hepatic cholesterol levels were similar among all groups except the ethanol-fed LE rats, in which the mean level was significantly higher than in all other groups except SD controls (Figure 1(f)).

**Figure 2:**
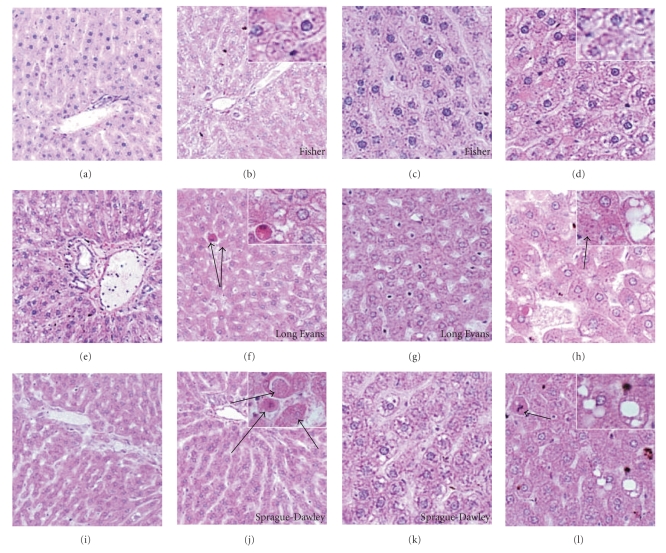
*Rat strain differences in susceptibility to alcohol-induced steatohepatitis*: histological sections of liver were stained with (a)–(l) H&E. Photomicrographs depict livers from (a, c, e, g, i, k) control or (b, d, f, h, j, l) ethanol-exposed (a)–(d) FS, (e)–(h) LE, or (i)–(l) SD rats. Note more prominent microsteatosis (vacuoles) and apoptosis (arrows) in ethanol-fed LE and SD compared with FS rats, and in ethanol-fed relative to control rats. Also, note microsteatosis in LE control livers (g). Original magnifications, Panels ((a), (b), (e), (f), (i), (j)): 80x, Panels ((c), (d), (g), (h), (k), (l)): 400x, Insets:650x.

**Figure 3:**
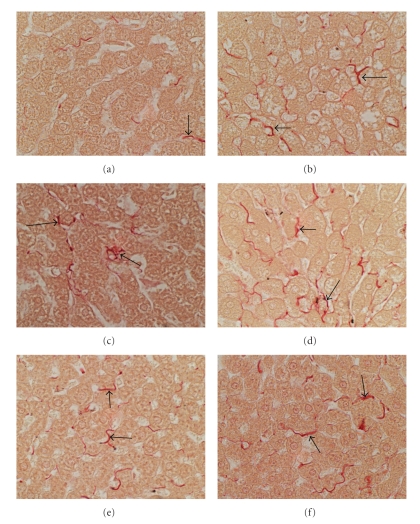
*Rat strain differences in susceptibility to alcohol-induced hepatic fibrosis*: histological sections of liver were stained with Sirius red to detect collagen. Photomicrographs depict livers from (a, c, e) control or (b, d, f) ethanol-exposed (a), (b) FS, (c), (d) LE, or (e), (f) SD rats. Note more prominent peri-hepatocyte Sirius red-positive collagen fibrils in ethanol-exposed livers and more extensive labeling in LE compared with FS and SD ethanol-fed rats. Original magnification: 650x.

Histopathological studies demonstrated the expected regular chord-like architecture with minimal steatosis or inflammation in control FS and SD livers, and mild microvesicular steatosis in control LE livers (Figure 2). Chronic ethanol feeding produced subtle increases in hepatocellular macrosteatosis with rare foci of lymphomononuclear inflammation in FS rats, but conspicuously increased mixed patterns of micro- and macrosteatosis with foci of inflammation and apoptosis in SD rats. LE rats had the most pronounced histopathological responses to chronic alcohol exposure characterized by increased disorganization of hepatic chord architecture, prominent micro- and macrosteatosis, and conspicuous foci of inflammation and necrosis or apoptosis. Sirius red-stained sections of liver revealed minimal collagen fibril deposition in FS controls, but conspicuously more abundant labeling of coarser peri-hepatocyte fibrils in LE and SD controls (Figures 3(a), 3(c), and 3(e)). All 3 strains exhibited increased peri-hepatocyte collagen deposition following 8 weeks of ethanol feeding, but the degrees were greater in LE and SD compared with FS rats (Figure 3). In addition, LE livers exhibited “chickenwire fibrosis” characterized by a trabecular or mesh-like pattern of fibrosis with collagen fibrils completely surrounding single hepatocytes (Figure 3(d)). 

### 3.3. Effects of Strain and Ethanol on Inflammatory Mediators

**Figure 4:**
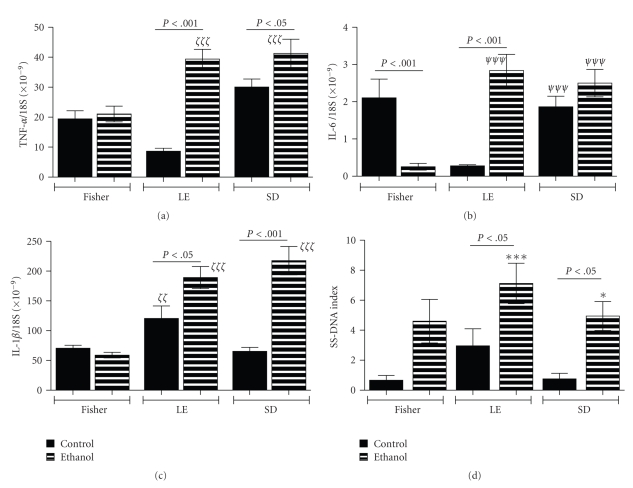
*Effects of strain and ethanol exposure on proinflammatory cytokine gene expression and DNA damage in liver*: qRT-PCR was used to measure (a) TNF-*α*, (b) IL-6, and (c) IL-1*β* expression, with results normalized to 18S rRNA. (d) Histological sections of liver were immunostained to detect single-stranded (SS) DNA as an index of nuclear DNA damage. Graphs depict the mean ± S.E.M. percentages of labeled hepatocyte nuclei (SS DNA Index). Intergroup comparisons were made using ANOVA with the post hoc Bonferroni multiple comparisons test of significance. Significant *P*-values are indicated within the panels. In addition, *P < .05 and ***P < .001 relative to FS Control; *ζ* *ζ* *P* < .01 and *ζ* *ζ* *ζ* *P* < .001 relative to FS control and ethanol fed rats; *ψ* *ψ* *ψ* *P* < .001 relative to FS ethanol-fed rats.

 To assess the effects of strain and chronic ethanol exposure on cytokine expression, we used qRT-PCR to measure interleukin-6 (IL-6), IL-1*β*, and tumor necrosis factor-alpha (TNF-*α*) mRNA levels (Figures 4(a)–4(c)). These proinflammatory cytokines were investigated because of their known roles in steatohepatitis due to chronic alcohol exposure or metabolic syndrome [32, 33]. In addition, we quantified DNA damage in histological sections by immunostaining for single-stranded DNA nicks and performing image analysis to determine the percentages of labeled nuclei (Figure 4(d)). In FS rats, the mean levels of TNF-*α* and IL-1*β* expression were unchanged by chronic ethanol feeding, whereas IL-6 expression was significantly reduced. In LE rats, chronic ethanol feeding significantly increased expression of all 3 cytokines relative to the pair-fed controls. In SD rats, chronic ethanol feeding significantly increased TNF-*α* and IL-1*β* but not IL-6 expression relative to control. Among ethanol-fed rats, the LE and SD strains had similar and significantly higher hepatic expression of all 3 cytokines relative to ethanol-fed FS rats. In addition, chronic ethanol feeding significantly increased the single-stranded DNA labeling indices in both LE and SD relative to corresponding and FS control livers. In the FS strain, single-strand DNA labeling indices were increased by ethanol exposure, but the difference from control did not reach statistical significance due to broad variability in responses.

### 3.4. Effects of Strain and Chronic Ethanol Exposure on Alcohol-Metabolizing Enzymes

**Figure 5:**
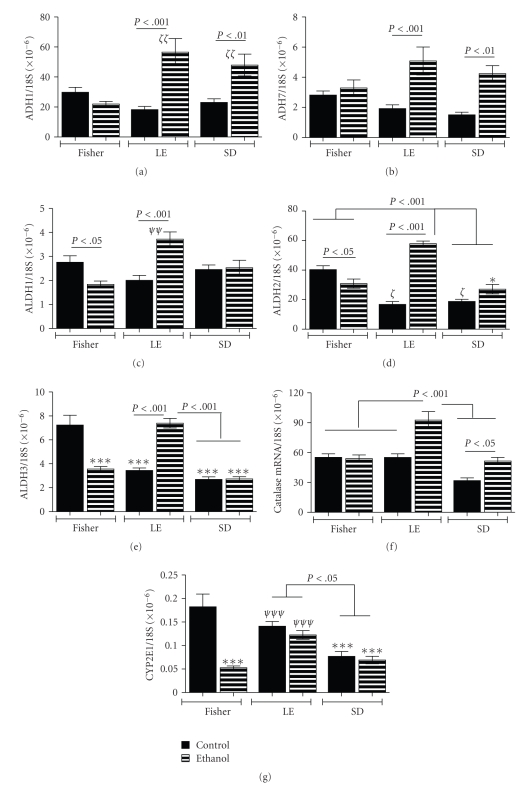
*Effects of strain and ethanol Exposure on alcohol metabolizing gene expression in liver*: qRT-PCR assays were used to measure (a), (b) alcohol dehydrogenases (ADH) 1 and 7, (c)–(e) aldehyde dehydrogenases (ALDH) 1-3, (f) Catalase, and (g) CYP2E1 gene expression. Graphs depict the mean ± S.E.M. levels of gene expression normalized to 18S rRNA. Intergroup comparisons were made using ANOVA with the post hoc Bonferroni multiple comparisons test of significance. Significant *P*-values are indicated within the panels. In addition, *P < .05 and ***P < .001 relative to FS Control; *ζ* *P* < .05, *ζ* *ζ* *P* < .01 relative to FS control and ethanol fed rats; *ψ* *ψ* *P* < .01 and *ψ* *ψ* *ψ* *P* < .001 relative to FS ethanol-fed rats.

 Ethanol is metabolized to acetaldehyde by alcohol dehydrogenases (ADH) and catalase, and from acetaldehyde to carbon dioxide and water via aldehyde dehydrogenase (ALDH) and CYP2E1 [34]. To determine if strain, that is genetic background, modulates liver capacity to metabolize ethanol, we measured ADH, ALDH, catalase, and CYP2E1 expression in liver by qRT-PCR. Exploratory studies showed that rat livers express ADH1, ADH7, ALDH1-ALDH3, catalase, and CYP2E1; therefore, our analyses were confined to these genes. Control rats had similar mean levels of hepatic ADH1 and ADH7 across the strains. Chronic ethanol exposure significantly increased ADH1 and/or ADH7 mRNA levels in LE and SD livers, but not FS livers (Figures 5(a) and 5(b)). 

Among controls, ALDH1 expression was similar in FS, LE, and SD rats, while ALDH2 and ALDH3 expressions were significantly lower in LE and SD relative to FS rats. In addition, catalase and CYP2E1 expression were significantly lower in SD relative to FS controls. With regard to chronic ethanol feeding, for the FS strain, the mean levels of ALDH1, ALDH3, and CYP2E1 were significantly reduced, while ALDH2 and catalase were unchanged relative to control (Figures 5(c)–5(g)). For the LE strain, chronic ethanol feeding significantly increased ALDH1, ALDH2, ALDH3, and catalase mRNA relative to control, but did not affect CYP2E1 expression. Within the SD strain, chronic ethanol exposure significantly increased catalase expression relative to control, but had no effect on hepatic expression of ALDH1, ALDH2, ALDH3, or CYP2E1. Therefore, LE rats were distinguished from FS and SD rats by their broad upregulation of genes that control rates of alcohol and acetaldehyde metabolism following chronic ethanol exposure. Among SD rats, the ethanol-stimulated increases in ADH and catalase, without corresponding increases in ALDH expression may reflect an increased tendency to accumulate acetaldehyde in liver. The significant ethanol-mediated downregulation of CYP2E1 in FS could represent a cytoprotective response, as CYP2E1 promotes oxidative stress in the context of ethanol exposure, and high levels of the enzyme are cytotoxic to liver cells [35, 36].

### 3.5. Effects of Strain and Ethanol Exposure on Insulin and IGF Receptor Binding

**Figure 6:**
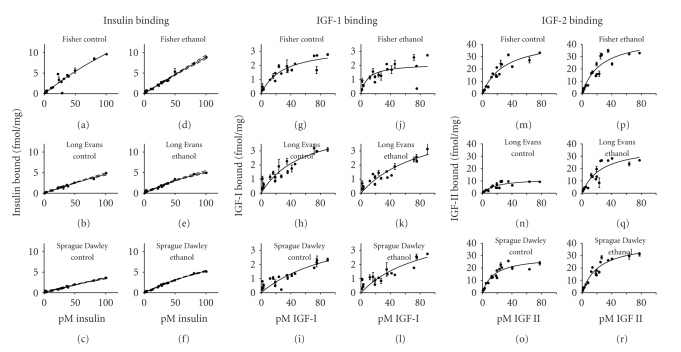
*Effects of strain chronic ethanol exposure on insulin, IGF-1, and IGF-2 receptor binding*: competitive saturation binding assays were used to measure specific insulin, IGF-1, and IGF-2 receptor binding with [^125^I] labeled insulin, IGF-I, or IGF-2 in the presence or absence of cold ligand. Radiolabeled bound ligand was harvested onto GF/C filter plates and measured in a TopCount. Specific binding curves (mean ± S.D.) are depicted for (a)–(f) insulin, (g)–(l) IGF-I, or (m)–(r) IGF-2 receptors in FS (a, g, m), LE (b, h, n), and SD (c, i, o) controls or FS (d, j, p), LE (e, k, q), and SD (f, l, r) ethanol-exposed livers. Statistics are shown in Table 3.

Since one of the major effects of chronic ethanol exposure is hepatic insulin resistance leading to impairments in energy metabolism, repair, and regeneration [12, 13, 37–40], we assessed the influence of genetic strain on insulin and IGF receptor binding in relation to chronic ethanol exposure. We performed competitive saturation binding assays and calculated the BMAX (top level binding) and Kd (binding affinity) for each group (Figure 6 and Table 3). Among controls, the BMAXs for insulin receptor binding were not distinguished by strain, whereas with regard to IGF-1, the BMAXs were significantly higher, and for IGF-2, they were significantly reduced in SD and LE relative to FS rats. The Kds for insulin and IGF-1 receptor binding were similar among the control groups whereas for IGF-2, the Kds were significantly lower (increased affinity) in SD and LE relative to FS livers. 

In FS rats, chronic ethanol feeding had no significant effect on the BMAX of insulin, IGF-1, or IGF-2 receptor binding, or the Kd for insulin or IGF-2 receptor binding. However, chronic ethanol feeding did significantly reduce the Kd, that is, it increased binding affinity for the IGF-1 receptor in FS rat livers. In SD and LE rats, the main effect of ethanol was to significantly reduce both the BMAX and Kd for insulin receptor binding relative to the levels observed in pair-fed controls, and both control and ethanol-fed FS rats (Table 3). Although ethanol did not significantly alter either the BMAX or Kd for IGF-1 receptor binding in LE and SD rats, each of these indices was significantly higher than in control and ethanol-fed FS rats. Paradoxically, chronic ethanol feeding in LE and SD rats significantly increased the BMAX and Kd for IGF-2 receptor binding relative to pair-fed controls. Consequently, the ethanol-associated IGF-2 receptor BMAXs and Kds were similar in LE, SD, and FS rat livers (Table 3). 

### 3.6. Consequences of Chronic Ethanol Consumption on Hepatic Gene Expression in Different Rat Strains

**Figure 7:**
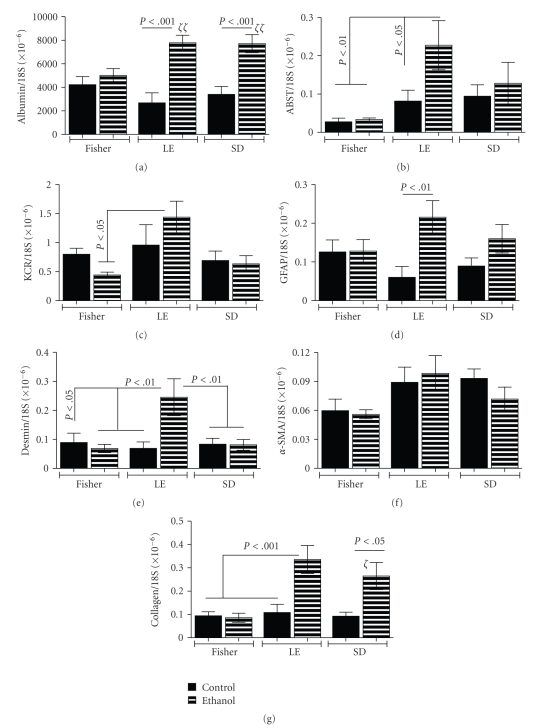
*Effects of strain and ethanol exposure on hepatic gene expression*: qRT-PCR assays were used to measure (a) albumin, (b) asialobiliary salt transporter (ABST), (c) Kupffer cell receptor gene, (d) glial fibrillary acidic protein (GFAP), (e) desmin, (f) *α*-smooth muscle actin (*α*-SMA), and (g) collagen, with levels normalized to 18S rRNA. Graphs depict the mean ± S.E.M. levels of gene expression. Intergroup comparisons were made using ANOVA with the post hoc Bonferroni multiple comparisons test of significance. Significant *P*-values are indicated within the panels. In addition, *ζ* *P* < .05, *ζ* *ζ* *P* < .01 relative to FS control and ethanol fed rats.

 We used qRT-PCR assays to examine strain and ethanol effects on gene expression corresponding to specific cell types and functions in liver (Figure 7). Albumin mRNA, which marks hepatocyte function, was expressed at similar levels in control FS, LE, and SD rats. Chronic ethanol feeding had no effect on albumin expression in FS rats, but significantly increased the levels in LE and SD rats (Figure 7(a)). Apical sodium-dependent bile acid transporter (ABST) marks bile duct epithelial cell function. The lowest ABST expression was measured in FS control and ethanol exposed livers. Among controls, there were no significant differences in the mean levels of ABST. Chronic ethanol exposure significantly increased ABST expression in LE, but not FS or SD rats. Kupffer cell glycoprotein receptor (KCR) marks resident macrophage abundance. Although the highest levels of KCR expression were observed in LE rats, the intergroup differences were not statistically significant. Glial fibrillary acidic protein (GFAP) is expressed in stellate cells. Alpha-smooth muscle actin (*α*-SMA) and desmin, an extracellular matrix product of stellate cells, report activated stellate cell function. The control groups had similar mean hepatic expression levels of GFAP, desmin, and *α*-SMA. Chronic ethanol feeding significantly increased GFAP and desmin expression only in LE rats. Otherwise, hepatic mRNA levels of GFAP, desmin, and *α*-SMA were not significantly modulated with respect to rat strain or ethanol exposure. Finally, collagen gene expression, which reflects fibrogenesis, was similarly low level in all control groups, and in ethanol-fed FS rats. However, in LE and SD rats, chronic ethanol feeding significantly increased pro-alpha-2(1) collagen (COL1a2) gene expression in liver. Therefore, chronic ethanol feeding significantly increased expression of albumin, ABST, desmin, GFAP, and COL1a2 in LE rats, and albumin and COL1a2 in SD rats. In contrast, in FS rats, ethanol had no significant effect on the expression levels of any of the hepatic genes examined.

**Figure 8:**
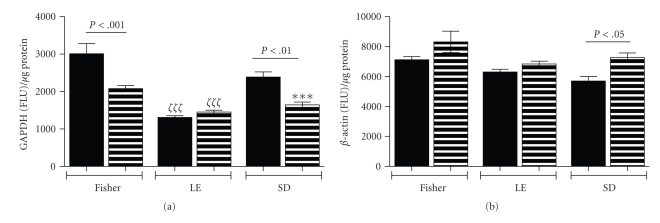
*Effects of strain and ethanol exposure on hepatic expression of glyceraldehyde-3-phosphate dehydrogenase, an insulin-responsive protein*: (a) GAPDH and (b) *β*-actin were measured in liver by ELISA. Graphs depict mean ± S.E.M of results. Intergroup comparisons were made using ANOVA with the post hoc Bonferroni multiple comparisons test of significance. Significant *P*-values are indicated within the panels. In addition, ***P < .001 relative to FS Control and *ζ* *ζ* *ζ* *P* < .001 relative to FS control and ethanol-fed rats.

We measured GAPDH immunoreactivity by ELISA to assess the effects of strain and ethanol exposure on the expression of an important insulin-responsive gene. We simultaneously measured *β*-actin immunoreactivity as a negative control (Figure 8). Among controls, GAPDH immunoreactivity was the highest in FS, followed by SD livers. In LE livers, basal GAPDH expression was significantly lower than in FS control livers. Chronic ethanol feeding significantly suppressed GAPDH expression in FS and SD, but not LE rats. Nonetheless, following chronic ethanol exposure, the mean levels of GAPDH were significantly higher in FS than LE or SD livers. The mean hepatic levels of *β*-actin were relatively similar among the 3 strains, irrespective of ethanol feeding. However, *β*-actin expression was significantly higher in ethanol exposed relative to control SD livers. 

## 4. Discussion

This study examined the effects of strain, that is, genetic background on an array of host factors related to alcohol metabolism, inflammation, and insulin/IGF signaling to determine if baseline or ethanol-induced differences predict proneness to alcohol-mediated liver injury. This work was inspired by findings in a previous study demonstrating that LE rats exhibited conspicuous alcohol-mediated hepatitis after 6–8 weeks of chronic exposure [12, 14, 22], whereas in other models, significant liver injury mainly occurs when ethanol exposure is combined with other injurious treatments [41, 42]. Moreover, empirical studies suggested that FS and SD rats were relatively insensitive to ethanol's effects on liver compared with LE rats. Therefore, we generated in vivo models of chronic ethanol feeding in which rats were pair-fed for 8 weeks with liquid diets containing 0% or 37% ethanol by caloric content. The expectation was that this type of investigation would eventually lead to the identification of biomarkers that could be applied to humans, and also help develop therapeutic approaches which target factors that increase proneness to progressive alcohol-related liver disease. 

The main findings in this study were that (1) there were clear strain differences concerning susceptibility to alcohol-mediated liver injury and steatohepatitis that were not attributable to differences in blood alcohol levels; (2) postexposure hepatic insulin resistance, characterized by reduced insulin receptor binding and insulin responsive gene expression, for example, GAPDH, correlated with enhanced susceptibility to alcohol-induced liver injury; (3) ethanol-induced increases in proinflammatory and pro-fibrogenic genes correlated with severity of steatohepatitis; (4) resistance to chronic alcohol-induced liver injury in FS rats was associated with minimal or negative (inhibitory) modulation of alcohol-metabolizing genes, particularly ADH1 and CYP2E1. Of note is that downregulation of ADH1 would serve to reduce acetaldehyde generation while reduced CYP2E1 would be protective against oxidative injury in the liver [35, 43]. Moreover, recent studies showed that chemical inhibition of CYP2E1 and other alcohol metabolizing enzymes reduces alcoholic steatohepatitis by decreasing triglyceride accumulation and proinflammatory cytokine levels in liver [44]. In essence, the FS strain appears to harbor an endogenously (genetically) favorable physiological state that protects the liver from alcohol-mediated injury, and utilizes mechanisms that could be exploited for therapeutic gains.

 Despite similar mean blood alcohol levels, histopathologic studies demonstrated that LE rats developed chronic alcohol-induced liver injury characterized by micro- and macrosteatosis, multifocal lymphomononuclear inflammation and necrosis/apoptosis, increased single-strand DNA labeling, that is DNA damage, and loss of the regular chord-like architecture. Livers of ethanol fed FS rats exhibited minimal injury and steatosis with relative preservation of hepatic chord architecture, whereas in SD rats, chronic ethanol feeding produced levels of steatohepatitis that were intermediate between LE and Fisher rats. Correspondingly, the serum ALT levels and degrees of triglyceride and cholesterol accumulation in liver were highest in ethanol-fed LE, followed by SD rats. Moreover, proinflammatory cytokine gene expression was sharply increased by chronic ethanol exposure in LE and SD, but not FS rats. 

Since blood alcohol levels were similar in the 3 strains, the strain-related adverse effects of ethanol most likely mark inherent (genetic) differences in susceptibility to alcohol-mediated liver injury. Of particular note is that we detected several baseline (nonethanol exposure related) abnormalities consisting of higher levels of serum ALT, hepatic cholesterol, DNA damage, and IL-1*β* mRNA, and lower levels of insulin receptor binding (BMAX) and affinity (higher Kd), and insulin responsive gene expression (GAPDH), consistent with insulin resistance, in LE compared with FS rats who proved to be fairly resistant to ethanol-mediated liver injury. This suggests that individuals at increased risk for developing significant and possibly progressive alcohol-related liver disease may manifest evidence of low-level ongoing hepatocellular injury, inflammation, and insulin resistance, together with increased cholesterol content in liver, and therefore could potentially be identified prior to significant alcohol exposure. 

Our studies also showed that the ethanol-induced expression levels of multiple genes that regulate alcohol metabolism, including CYP2E1, were correlated with severity of steatohepatitis, that is, they were highest in LE, intermediate and variable in SD, and lowest in FS rats. To some extent, this result is at variance with a recent report by Ciuclan et al. in which downregulation of ADH by TGF-*β*1 was correlated with increased hepatocellular injury [45]. However, the differences might be explained by the fact that the responses were demonstrated using relatively short-term isolated hepatocyte cultures, and the overall goal of their experiment was to examine how TGF-*β*1 enhances ethanol-induced hepatotoxicity. Therefore, TGF-*β*1 was the main independent variable in the paper by Ciuclan et al., whereas chronic ethanol exposure was the primary independent variable in the present work. Finally, the findings herein suggest that differences in response to ethanol may vary according to genetic factors, including strain or species. 

The differences observed among the 3 strains with respect to ethanol's effects on alcohol-metabolizing enzyme gene expression could reflect inherent differences in alcohol metabolism efficiency, with higher levels of gene expression being required to detoxify ethanol in LE than in FS rats. Consequences of inefficient alcohol metabolism would include increased acetaldehyde build-up and oxidative stress. In addition, the relatively higher levels of CYP2E1 expression in ethanol-exposed LE compared with ethanol-exposed FS livers may have contributed to the increased severity of steatohepatitis in LE rats because high levels of CYP2E1 promote oxidative stress and reduce fatty acid oxidation via inhibition of PPAR-*α* expression, and thereby enhance ethanol-induced hepatic steatosis [43]. Correspondingly, the ethanol-induced downregulation of CYP2E1 expression in the FS strain could represent an inherent cytoprotective response, abrogating the cytotoxic effects of ethanol on liver cells [35, 36]. 

To better understand the contributions of insulin and/or IGF resistance in the pathogenesis of alcohol-induced chronic liver disease, we measured insulin, IGF-1, and IGF-2 receptor binding. Those investigations mainly demonstrated reduced insulin receptor binding in ethanol-exposed LE and SD relative to FS livers. Likewise, GAPDH, which is regulated by insulin [46], was expressed at lower levels in ethanol-exposed SD and LE compared to FS livers. The finding of reduced GAPDH expression in control LE rats further suggests impairments in hepatic insulin signaling in this strain at baseline, despite relatively preserved insulin receptor binding. These results corroborate the concept that chronic ethanol exposure causes hepatic insulin resistance. Furthermore, the findings suggest that endogenous/genetic factors dictate the degree to which chronic ethanol exposure will likely cause steatohepatitis and hepatic insulin resistance. 

With regard to IGF-1 receptor binding, the major influence of strain was to significantly lower the binding affinity in SD and LE rats, with or without chronic ethanol exposure. Since IGF-1 is an important regulator of hepatocellular growth and repair, IGF-1 resistance would likely contribute to impaired regenerative and reparative capacity of the liver. The finding that SD and LE controls had significantly lower BMAXs for IGF-2 receptor binding in liver relative to FS controls supports the notion that endogenous inter-strain differences pertain to both basal and ethanol-induced differences in insulin/IGF signaling mechanisms in the liver. On the other hand, the ethanol-associated normalization of IGF-2 receptor BMAX in SD and LE livers is of interest in light of the coexisting impairments in insulin and IGF-1 receptor binding, as IGF-2 can crosstalk with insulin and IGF-1 signaling pathways [47]. Consequently, the ethanol-associated enhancements of IGF-2 receptor binding could represent compensatory responses that aided in maintaining survival and metabolic functions in ethanol-exposed LE and SD livers. 

Finally, in LE rats, chronic ethanol exposure significantly increased expression of ABST, GFAP, desmin, and collagen, indicating that bile duct epithelium and stellate cells were more activated, and that genes responsible for hepatic fibrosis, that is, desmin and collagen, were up-regulated. In our models, *α*-SMA, the most widely used marker of early stellate cell activation which marks transition from quiescence to a contractile myofibroblast phenotype [48, 49], was expressed in livers from all groups. Although *α*-SMA mRNA levels were not significantly increased by chronic ethanol exposure, 3 other indices of the pro-fibrogenic state were increased in LE rats, correlating with the increased Sirius Red staining of collagen in liver. Previously it was shown that increased GFAP expression marks activated stellate cells in rat liver [50], and increased levels correlate with hepatic fibrosis in human livers [51], consistent with the findings herein. Moreover, cells other than stellate cells, including portal fibroblasts and bone marrow derived cells contribute to hepatic fibrosis [48]. Activation of stellate and other extracellular matrix producing cells with attendant increased expression of pro-fibrogenesis genes most likely reflects a tendency for the alcohol-induced steatohepatitis to progress to hepatic fibrosis and liver dysfunction. Although stellate cell activation and fibrogenesis are known consequences of chronic alcohol abuse in humans [52–54], our results link genetic factors and ethanol-mediated insulin/IGF-1 resistance to severity of steatohepatitis and proneness to develop progressive alcoholic liver disease. 

An important and novel conclusion from these studies is that low levels of chronic injury, inflammation, and insulin resistance in the “normal” or baseline state of liver function could play a critical role in predisposing individuals to alcohol-induced chronic liver disease. A second point is that ethanol-induced stimulation of alcohol-metabolizing enzymes, or downregulation of CYP2E1, distinguishes the 3 rat strains according to their differential susceptibilities to chronic alcohol-mediated liver injury. Third, the degrees to which GFAP, desmin, and collagen 1 expression, that is indices of fibrogenesis, were increased by chronic ethanol exposure correlated with inherent strain sensitivity to ethanol-induced liver injury. These results suggest that future investigations should focus on both basal and ethanol-induced abnormalities in liver function including insulin responsiveness, cell turnover, inflammation, alcohol metabolizing enzyme gene expression, and pro-fibrogenesis pathways. Furthermore, our findings suggest that additional biomarkers that report mild insulin resistance, injury, and proinflammatory states in liver may help predict long-term adverse responses to alcohol, and possibly other drugs that utilize alcohol detoxification pathways. 

## Figures and Tables

**Table 1:** Primer pairs for quantitative RT-PCR assays.

Primer		Sequence (5′ → 3′)	Position	Size (bp)
Desmin	For	ACCTGCGAGATTGATGCTCT	1069	206
Desmin	Rev	ACATCCAAGGCCATCTTCAC	1274	
COL1a2	For	ACCTCAGGGTGTTCAAGGTG	1632	222
COL1a2	Rev	CGGATTCCAATAGGACCAGA	1844	
*α*-SMA	For	TGTGCTGGACTCTGGAGATG	516	148
*α*-SMA	Rev	GAAGGAATAGCCACGCTCAG	663	
ASBT	For	GCATTGGCATTTCTCTGGTT	598	181
ASBT	Rev	GGTTCAATGATCCAGGCACT	778	
GFAP	For	GGTGGAGAGGGACAATCTCA	433	215
GFAP	Rev	CTCGAACTTCCTCCTCATGG	647	
ALB	For	CTTCAAAGCCTGGGCAGTAG	702	188
ALB	Rev	TGGAGATAGTGGCCTGGTTC	889	
KCR	For	TCACAAATGCTGTGGACCAT	1427	161
KCR	Rev	GTCTTCACGCTCTCCGTTTC	1587	
ADH-1	For	TGCTCCGTGCTGGAAAGAGTATCC	1138	117
ADH-1	Rev	TAAGGTTGTGATGTGGCTGGCG	1254	
ADH-7	For	CAATGCTGCTTTTCACTGGA	971	234
ADH-7	Rev	AGAACACCCAGCTCTCTGGA	1204	
ALDH-1	For	ATCTGCCATGTGGAAGAAGG	173	216
ALDH-1	Rev	CAAGTACGCATTGGCAAAGA	388	
ALDH-2	For	GACCTGGACAAGGCCAATTA	1391	193
ALDH-2	Rev	TCTTCTGTGGCACTTTGACG	1583	
ALDH-3	For	CTGATTGCTGAGGTTCCTGTTAGG	1914	119
ALDH-3	Rev	GGATGTTTAGACTGAGAGCCGACTC	2032	
Catalase	For	ATACGAAGGTGTTGAATGAGGAGG	1369	63
Catalase	Rev	TCAGGTGGTTGGCAATGTTCTC	1431	
CYP2E1	For	GTGTTCACACTGCACCTTGG	211	69
CYP2E1	Rev	CACCTCCTTGACAGCCTTGT	279	
GAPDH	For	AGT GGG CAT CAA TGG ATT TGG	306	241
GAPDH	Rev	GGG GAT TTC CTT AGG TTC TTT GC	546	
IL-1*β*	For	CAG CAG CAT CTC GAC AAG AG	233	60
IL-1*β*	Rev	CTT CTC CAC AGC CAC AAT GA	292	
TNF-*α*	For	ATG TGG AAC TGG CAG AGG AG	26	84
TNF-*α*	Rev	AGA AGA GGC TGA GGC ACA GA	109	
IL-6	For	ATG TTG TTG ACA GCC ACT GC	116	51
IL-6	Rev	GTC TCC TCTCCG GAC TTG TG	166	

qPCR: quantitative polymerase chain reaction; ASBT: apical sodium dependent bile acid transporter; KCR: Kupffer cell glycoprotein receptor; GFAP: glial fibrillary acidic protein; COL1a2: pro-alpha-2(I) collagen; ALB: albumin; *α*-SMA: alpha smooth muscle actin; ADH: alcohol dehydrogenase; ALDH: aldehyde dehydrogenase; CYP2E1: cytochrome P450 2E1; GAPDH: glyceraldehyde-3-phosphaye dehydrogenase; IL: interleukin 1; TNF: tumor necrosis factor.

**Table 2:** Weight gain after 8 weeks on isocaloric liquid diets*.

	FS Control	FS Ethanol	LE Control	LE Ethanol	SD Control	SD Ethanol
# Rats/Group	12	14	12	13	12	13

Wt Gain (gm)	82.68	55.49^c,d^	75.05 ^e^	64.18 ^b^	104.4	90.88
± S.E.M.	5.35	3.83	6.49	6.92	7.93	6.86

% Wt Gain	30.63	21.16 ^a,b^	21.04 ^a,b^	19.59 ^a,b^	34.75	30.02
S.E.M.	1.98	1.42	1.77	2.32	2.73	2.34

*Adult Fisher (FS), Long Evans (LE), and Sprague-Dawley (SD) rats were pair-fed with isocaloric control or ethanol-containing liquid diets for 8 weeks, and were weighed weekly (See Section 2). Net mean body weight (grams) and mean percentage body weight gained over the 8 week period were calculated. Intergroup comparisons were made using two-way ANOVA and Tukey post hoc tests of significance. ^a^significantly lower than FS Control (*P* < .05); ^b^significantly lower than SD Control (*P* < .001) and SD Ethanol (*P* < .05); ^c^significantly lower than FS Control (*P* < .05); ^d^significantly lower than SD Control (*P* < .001) and SD Ethanol (*P* < .01); and ^e^significantly lower than SD Control (*P* < .05).

**Table 3:** Insulin and insulin like growth factor receptor saturation binding assay results for liver.

Receptor	FS-Control	FS-Ethanol	LE-Control	LE-Ethanol	SD-Control	SD-Ethanol
*Insulin*						
Bmax	46.61 ± 27.43	99.85 ± 60.68	43.41 ± 24.54	16.9 ± 2.98**	29.24 ± 14.42	18.94 ± 2.18***
Kd	380.7 ± 262.6	1028 ± 666.8	830.9 ± 508.7	228.3 ± 51.03***	716.6 ± 387.1	260.2 ± 37.18***
*R* ^2^	0.8888	0.9799	0.9743	0.972	0.9726	0.9907

*IGF-1*						
Bmax	3.35 ± 0.33	2.13 ± 0.24	5.11 ± 0.83**	5.42 ± 1.15***	5.13 ± 1.77***	5.02 ± 1.34***
Kd	29.45 ± 6.89	9.46 ± 4.20**	57.34 ± 17.23	88.34 ± 30.31	114 ± 58.96	89.7 ± 38.36
*R* ^2^	0.8684	0.5333	0.8156	0.8313	0.6977	0.735

*IGF-2*						
Bmax	45.00 ± 4.40	46.36 ± 5.04	12.13 ± 1.41***	38.59 ± 5.19	29.46 ± 2.15*	41.29 ± 3.17
Kd	30.28 ± 5.99	24.86 ± 5.87	18.74 ± 5.28***	25.51 ± 7.38	15.95 ± 3.01***	22.43 ± 3.88
*R* ^2^	0.9099	0.8685	0.8028	0.8131	0.9026	0.9197

Saturation binding assays were performed with liver tissue homogenates from Fisher 344 (FS), Sprague Dawley (SD), or Long Evans (LE) rats maintained on liquid diets containing 0% (control) or 37% ethanol (caloric content) for 8 weeks. The BMAX (top-level binding), Kd (affinity), and *R* ^2^ (correlation coefficient) were calculated using Prism 5 software. Intergroup comparisons of the one-site specific binding curves were made using extra sum of squares *F*-tests. For all groups, the df = 38.  *P = .0008; **P = .0002;  ***P < .0001 relative to corresponding FS control or ethanol-exposed group.
